# COVID-19 Associated Spontaneous Pneumothorax and Pneumopericardium: A Case Report

**DOI:** 10.7759/cureus.14861

**Published:** 2021-05-05

**Authors:** Jacques Bistre, Steven Douedi, Abbas Alshami, Jeffrey Ndove, Joseph Varon

**Affiliations:** 1 Research, Dorrington Medical Associates, Houston, USA; 2 Internal Medicine, Jersey Shore University Medical Center, Neptune City, USA; 3 Internal Medicine, Dorrington Medical Associates, Houston, USA; 4 Critical Care, United General Hospital, Houston, USA; 5 Critical Care, University of Texas Health Science Center at Houston, Houston, USA

**Keywords:** covid-19, coronavirus, pneumothorax, pneumomediastinum, pneumopericardium

## Abstract

Novel coronavirus 2019 (COVID-19) has been one of the largest and most devastating global pandemics of our time. There have been several complications of this disease that have also proven to be debilitating and deadly. While primarily affecting the respiratory system, some cases presented with uncommon complications such as pneumopericardium and spontaneous pneumothorax. We present a case of an elderly female diagnosed with COVID-19 found to have both spontaneous pneumothorax and pneumopericardium. She had a complicated hospital course and ultimately succumbed to her illness. While the pathogenesis of these conditions is not yet fully understood, further studies are needed to help clinicians develop treatment and prevention strategies to improve patient outcomes.

## Introduction

Coronavirus 2019 (COVID-19) became a public health international emergency in December 2019 leading to hundreds of thousands of deaths to date [1,2]. COVID-19, caused by an enveloped RNA virus, is known to cause severe pneumonia with complications that lead to acute respiratory distress syndrome (ARDS), cytokine storm, and disseminated intravascular coagulation (DIC) [1,3-5]. Several cases have reported spontaneous pneumomediastinum and pneumothorax secondary to COVID-19; however, the underlying pathophysiology remains to be understood and pneumopericardium still remains uncommon [5,6]. We present a case of an otherwise healthy elderly female diagnosed with COVID-19 found to have both pneumothorax and pneumopericardium on routine chest X-ray after intubation for hypoxia.

## Case presentation

**Figure 1:**
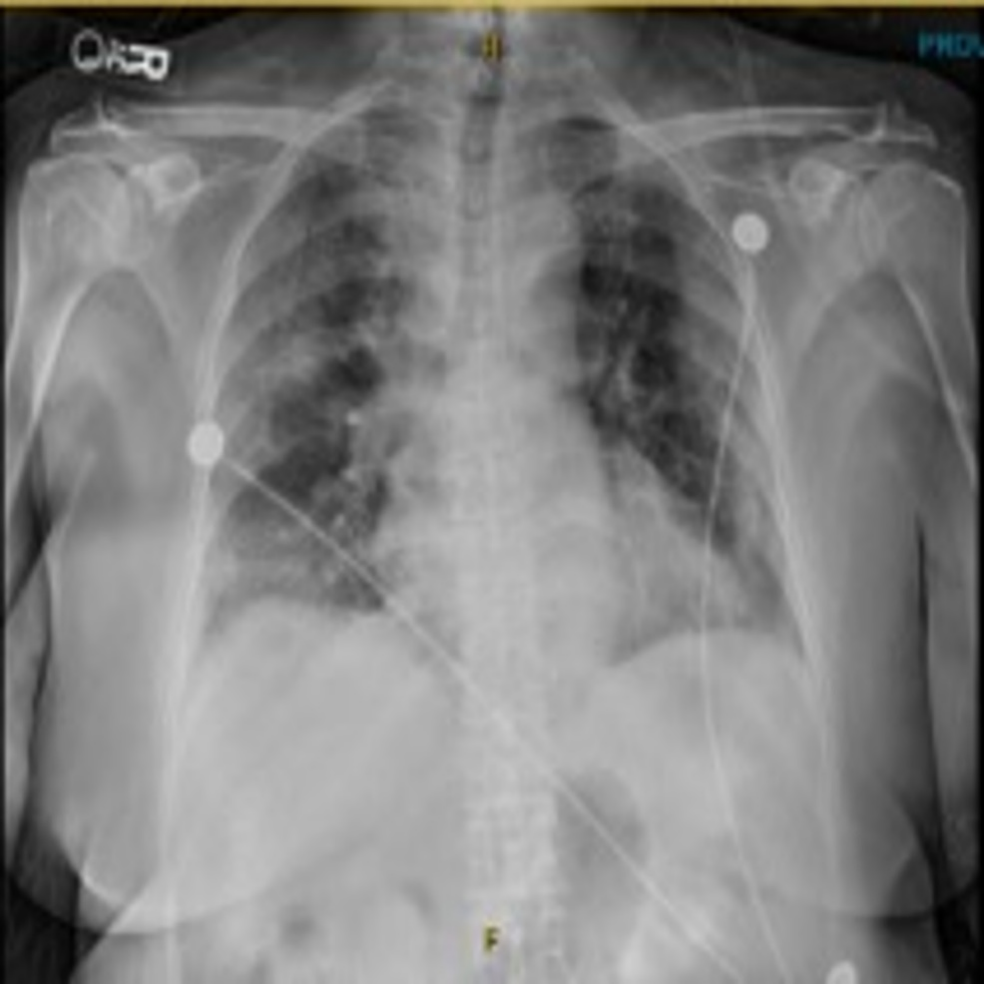
Chest X-ray on admission. Chest X-ray on admission showing bilateral ground-glass opacities consistent with COVID-19 infection.

A 70-year-old female with no significant medical history presented to the emergency department complaining of shortness of breath, palpitations on exertion for the last one week prior to arrival, and unquantified fever for the previous three days. Her vitals on admission were a heart rate of 91 beats per minute, respiratory rate (RR) of 20 breaths per minute, blood pressure of 119/64 mm Hg, the temperature of 100.3 degrees Fahrenheit, and oxygen saturation (SpO_2_) of 90% on room air. Physical examination was only remarkable for tachypnea and increased work of breathing for which she was placed on high flow oxygen via nasal cannula with a fraction of inspired oxygen (FiO_2_) of 85% and RR of 35 to maintain an oxygen saturation of 89%-90%. Laboratory findings on admission were unremarkable and she was diagnosed with COVID-19 infection through reverse transcription-polymerase chain reaction (RT-PCR). Chest X-ray was also obtained on admission showing bilateral ground-glass opacities consistent with COVID-19 infection (Figure 1). Four days after admission, her oxygen saturation dropped below 88% but her vitals otherwise remained stable. Due to persistent tachypnea despite high flow oxygen, she was switched to bilevel positive airway pressure (BiPap) with an inspiratory positive airway pressure of 12 and expiratory positive airway pressure of 6 and 100% FiO_2_.

**Figure 2:**
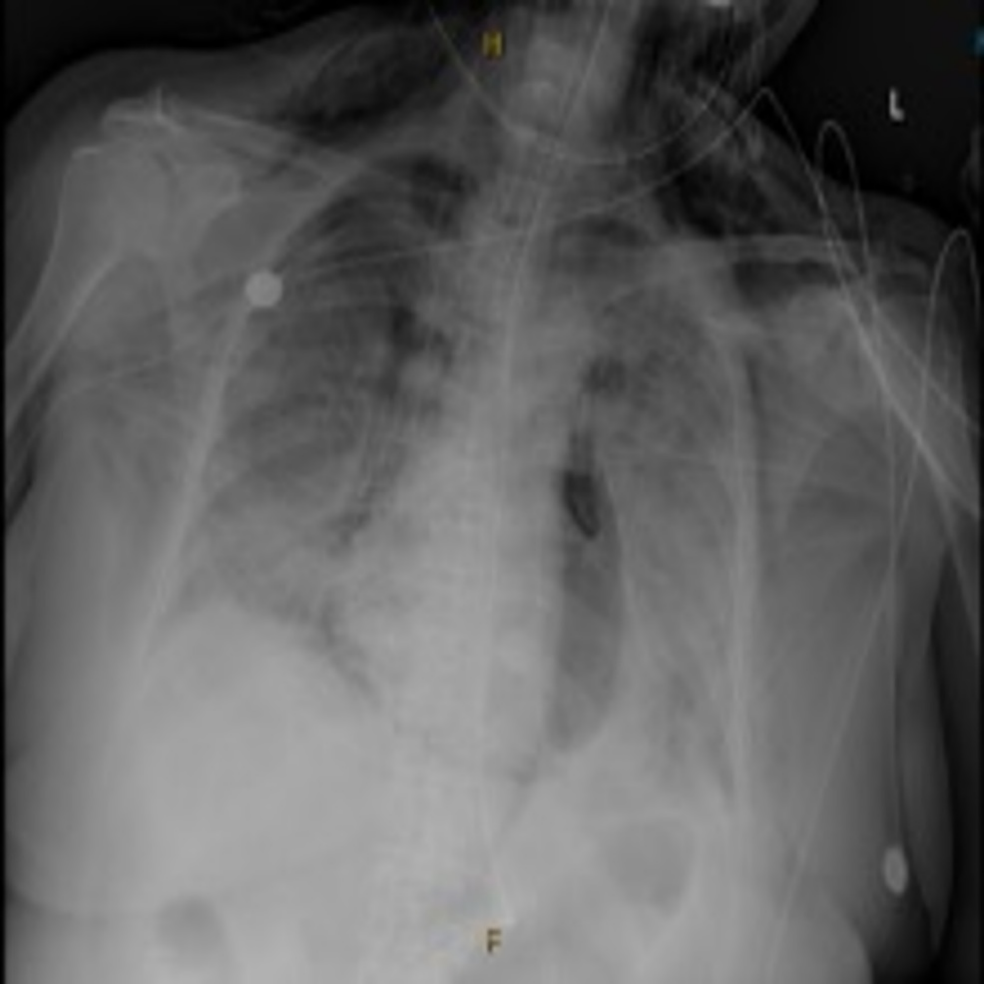
Post intubation chest X-ray. Post intubation chest X-ray showing both pneumothorax of the right lung and pneumopericardium.

Six days later, she began to desaturate again while on 100% FiO_2_ via BiPap (arterial blood gas at that time: pH 9.45, PCO_2_ 38.8, PO_2_ 60, HCO_3_ 27.5, and SpO_2_ 92%). She was encouraged to self-pronate but was unable due to discomfort. She was ultimately intubated due to persistent hypoxia and tachypnea and brought to the intensive care unit (ICU) for further management. Post intubation chest X-ray was performed showing both pneumothorax of the right lung and pneumopericardium which were not present prior to intubation (Figure 2). Mechanical ventilator settings at that time on pressure control mode with 25 cm H_2_O, positive end-expiratory pressure (PEEP) of 10 cm H_2_O, and FiO_2_ of 100%.

**Figure 3:**
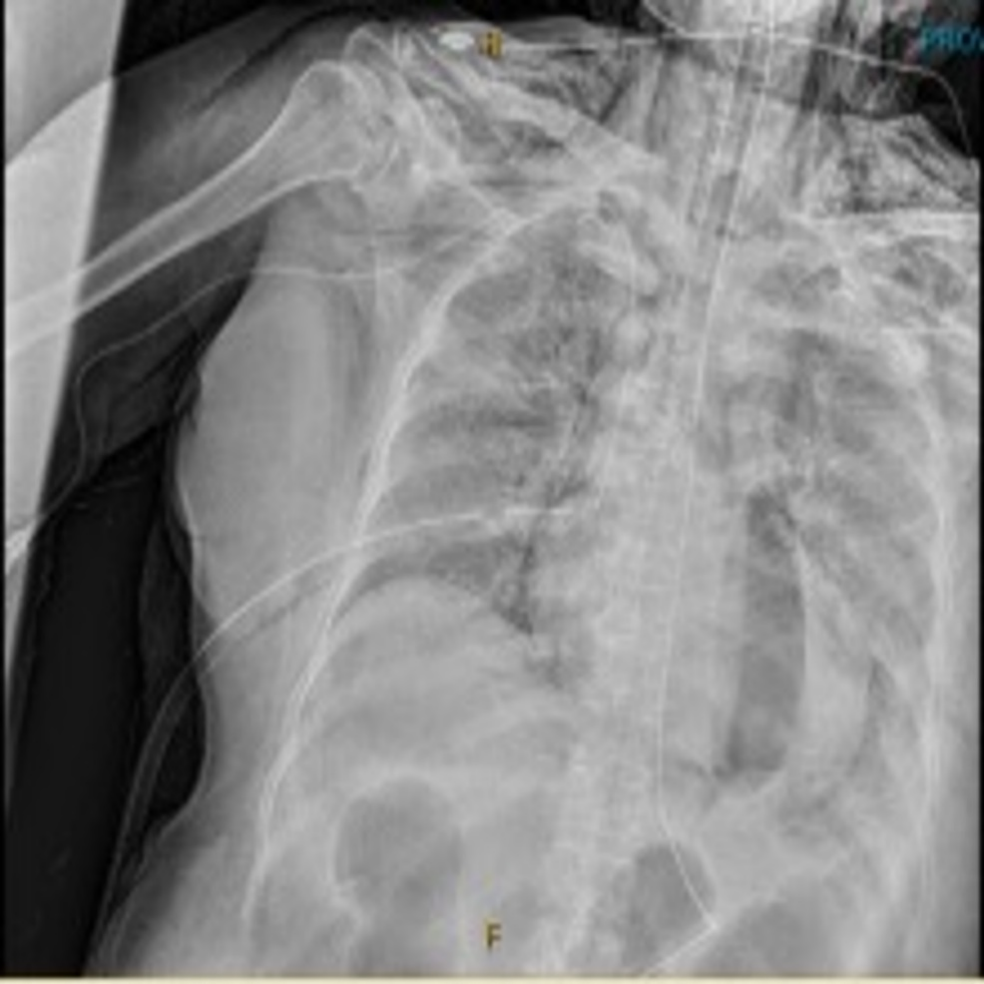
Chest X-ray after first chest tube. Chest X-ray showing persistent right pneumothorax despite initial chest tube placement requiring second chest tube.

After the discovery of the pneumothorax, a right-sided chest tube was emergently placed; however, the following day it was noted on chest X-ray that the right pneumothorax persisted and a second chest tube was placed which helped to resolve the pneumothorax (Figure 3). On day 18 of hospitalization, despite aggressive management in the ICU, she continued to have persistent hypoxemia. To decrease the oxygen demand of the body, especially the brain, it was decided to put the patient under therapeutic hypothermia, and she was cooled to 32 degrees Celsius. After 48 hours, the rewarming phase was started; however, due to a poor prognosis her family decided to begin hospice and comfort care and the patient passed away on day 21 of hospitalization.

## Discussion

Pneumothorax is defined as the presence of air in the pleural space [7]. Spontaneous pneumothorax has an incidence of 7.4 to 18 cases per 100,000 population each year in males and 1.2 to 6 cases per 100,000 population each year in females [7]. Symptoms primarily depend on the size of pneumothorax being asymptomatic when small but progressing to dyspnea and chest pain with decreased or absent breath sounds and hyper-resonance on percussion [7]. Pneumothorax can be due to communication between alveolar spaces and pleura, direct or indirect communication between the atmosphere and the pleural space, or the presence of gas-producing organisms in the pleural space [7]. Petersen et al. reported that peak airway pressure over 50 cm H_2_O is associated with an increased risk of alveolar rupture during mechanical ventilation [8]. There have also been correlations made between high peak airway pressure and the development of pneumothorax [8]. Although high PEEP had been reported to be associated with pneumothorax, several studies have found no such relationship [9-11]. In our case presentation, the patient was only on a PEEP of 10, which decreased the suspicion of mechanical ventilation as a cause for the pneumothorax.

Pneumomediastinum is defined as free air in the mediastinum and is classified as spontaneous pneumomediastinum (SPM) and secondary pneumomediastinum [12]. SPM tends to be benign and idiopathic while secondary can be the result of trauma, infections, esophageal, or tracheobronchial tree lesions [12]. According to Caceres et al., pneumomediastinum can develop without a triggering event and with no notable findings on chest radiography [12]. In the setting of COVID-19, there have been some reported cases of worsened patient outcomes when pneumomediastinum was present [6,9,10].

Pneumopericardium is defined as the presence of air in the pericardial cavity. It can be the result of blunt chest wall trauma in deceleration injury and as a rare complication of pericardiocentesis or positive pressure ventilation [13]. Lee et al. and others suggest that the physiopathology may be due to higher intrathoracic pressure where the air ruptured alveoli can dissect through the superior and anterior mediastinal part and hilum opening of the lungs and enter pericardium along venous sheaths, which have lower collagenous support [13,14]. Finding pneumopericardium is an unusual condition only found to occur in 1% of hospitalized patients with COVID-19 but has been associated with increased disease severity and poor clinical outcomes, as seen in our patient [5]. After excluding barotrauma due to elevated PEEP as the cause of this patient's condition, pneumopericardium and pneumothorax can both be spontaneous conditions seen in COVID-19 patients as other studies also suggested.

## Conclusions

Although associated with poor outcomes and uncommon, several published cases and our own experience emphasize the importance of early identification and management of these complications in affected patients. While the pathogenesis of these conditions is not yet fully understood in the setting of COVID-19 infection, further studies are needed to help clinicians develop treatment and prevention algorithms to improve patient outcomes.
